# Immunomodulatory functions of glutaminyl cyclases QPCTL and QPCT

**DOI:** 10.3389/fimmu.2026.1760809

**Published:** 2026-03-26

**Authors:** Hannah Elaine Smid, Jana Colotti, Sophia Nölp, Nike Sophia Arlt, Sophie Weber, Amelie Gumann, Stephan von Hörsten, Axel Karow, Wolfgang Schuh

**Affiliations:** 1 Department of Translational Immunology, Department of Medicine 3 – Rheumatology and Immunology, Nikolaus-Fiebiger Center for Molecular Medicine, Universitätsklinikum Erlangen, Friedrich-Alexander-Universität Erlangen-Nürnberg (FAU), Erlangen, Germany; 2 Department of Experimental Therapy, Preclinical Experimental Center, Universitätsklinikum Erlangen, Friedrich-Alexander-Universität Erlangen-Nürnberg (FAU), Erlangen, Germany; 3 Department of Paediatrics and Adolescent Medicine, Universitätsklinikum Erlangen, Friedrich-Alexander-Universität, Erlangen-Nürnberg (FAU), Erlangen, Germany

**Keywords:** glutaminyl cyclases, immune cells, IsoQC, pyroglutamate, QC, QPCT, QPCTL

## Abstract

Glutaminyl-peptide cyclotransferase (QPCT, QC) and its isoenzyme glutaminyl-peptide cyclotransferase-like protein (QPCTL, isoQC) are zinc-dependent enzymes that post-translationally catalyze the conversion of N-terminal glutamine or glutamate residues into pyroglutamate (pGlu). The pGlu modification impacts protein-protein interactions, enhances protein stability, and protects proteins from proteolytic degradation. QPCTL and QPCT differ in their subcellular localization, with QPCTL being retained in the Golgi apparatus and QPCT being active in secretory vesicles. Current research focuses on the impact of QPCTL-mediated pGlu formation in cancer and neurodegenerative disorders such as Alzheimer’s disease. In cancer, QPCTL is a promising immunotherapy target since QPCTL-mediated CD47 pyroglutamylation prevents macrophages from phagocytosing tumor cells. Moreover, QPCTL shapes the tumor microenvironment by modulating macrophage recruitment and polarization through modification of CCL2. However, QPCTL modulates Butyrophilins on tumor cells and thereby promote their detection and killing by γδ T cells. Hence, QPCTL significantly affects cancer progression, inflammatory processes, and immune regulation. These insights highlight QPCTL’s potential as a therapeutic target in oncology, metabolic diseases, and immune-mediated disorders. In this review, we highlight the role of QPCTL in tumor evasion and immune modulation. Moreover, we provide a comprehensive overview about predicted and validated substrates of QPCT/L and about the relevance of QPCT/L in various diseases.

## QPCTL and QPCT

Glutaminyl-peptide cyclotransferase-like protein (QPCTL, isoQC) is the isoenzyme of glutaminyl-peptide cyclotransferase (QPCT, QC). Both QPCTL and QPCT catalyze the conversion of N-terminal glutamine or glutamate in peptides and proteins into pyroglutamate through a zinc-dependent catalytic mechanism (1, 2). Between the two isoenzymes there is strong shared identity (45% between human QCs) and high conservation within the active site domains (3, 4). In the case of glutamine, glutaminyl cyclase activity leads to ammonia being released and a pyroglutamate lactam ring forming. Since glutamine can be protonated and charged at its N-terminus, the pGlu ring formation results in the removal of the positive charge from the N-terminal amino group. In case of glutamate, water is released as a pyroglutamate lactam ring is formed and the free carboxyl group is removed (5). Typically, the formation of the pGlu ring structure constitutes a mechanism to promote stability and prevent degradation by peptidases (6, 7).

Structural differences between QPCTL and QPCT determine their subcellular compartmentalization. QPCTL is retained within the Golgi apparatus by the N terminus membrane anchor and is highly dependent on the Conserved Oligomeric Golgi (COG) complex for intra-Golgi retrograde trafficking (4, 8). In contrast, QPCT has a signal sequence guiding the enzyme to the secretory pathway (4). When the membrane anchor of human QPCTL was removed and expressed in *Pichia pastoris*, QPCTL was secreted, however, it did not lead to glutaminyl cyclase activity in the media. Only after an N-glycosylation site similar to that in QPCT was introduced could glutaminyl cyclase activity be detected in the media (3).

The separate subcellular localization of QPCTL and QPCT allows for differential substrate conversion *in vivo*, despite identical substrate conversion *in vitro* (9). For example, QPCTL modifies CD47 and CCL2, while QPCT modifies amyloid-β (Aβ) and thyrotropin-releasing hormone (TRH). To better understand the regulation and signaling of glutaminyl cyclases, it is crucial to differentiate between the two isoenzymes in studies.

## QPCTL and QPCT in human and mouse

The human QPCTL gene on chromosome 19 encodes for two protein-coding variants through alternative splicing and is ubiquitously expressed throughout the body (10, 11). The longer variant consists of 382 amino acids, while the shorter variant only consists of 288 amino acids due to exon three (amino acids 118-211) being spliced out (10). However, the active site of QPCTL is predicted to be at amino acid 225 and 269, which suggests that the enzymatic activity is still functional in the shorter variant (12). The gene for QPCT in humans is located on chromosome 22 and encodes four protein-coding splice variants of 361, 284, 117, and 137 amino acids (10).

For both QPCTL and QPCT there is high conservation between the human and mouse sequences, however, there are limitations in the model due to different splicing variants. Mice only express one QPCTL protein encoding variant consisting of 383 amino acids and two QPCT protein encoding variants of 362 and 313 amino acids (10). As of yet, there has been no research published on any potential functional implications of the splicing variants.

## QPCT/L in hormonal regulation

The pGlu modification is crucial for the maturation and the bioactivity of various hormones, such as TRH and gonadotropin-releasing hormone (GnRH). TRH is a short peptide produced by the hypothalamus that stimulates thyroid-stimulating hormone released by the anterior pituitary gland (13). Prohormone processing of TRH in secretory granules is primarily QPCT-mediated since its production was only impacted in QPCT-deficient mice, but not QPCTL-deficient mice (9). The pGlu modification protects TRH from degradation by peptidases and modulates its activity (14, 15). GnRH is a short decapeptide that regulates the hypothalamic-pituitary-gonadal axis and controls the serum levels of sex hormones, such as estrogen, and thereby bone mass (16, 17). GWAS and SNP-based association studies identified QPCT as one of four candidate genes linked to bone mineral density (BMD), with specifically variant rs3770748 in intron 5 of the QPCT gene being associated with lower BMD (18, 19). Therefore, QPCT may be involved in osteoporosis pathogenesis as a modulator of GnRH stability and degradation (20). Moreover, pGlu modifications were also found in other hormones and neuropeptides such as neurotensin (21).

Within the intronic region of QPCTL, the rs2287019 C allele is correlated with increased BMI. Specifically, the allele is associated with a higher insulinogenic index, disposition index, LDL-cholesterol, and total cholesterol (22, 23). A potential explanation for this is that this variant has high linkage disequilibrium with the GIPR gene variants in the same locus, which is implicated in beta cell function and insulin secretion (22). Ren et al. found that indels in the QPCTL promoter region led to higher daily weight gain in chickens. QPCTL overexpression in chicken primary myoblasts led to higher proliferation and inhibited differentiation (24). Recently, a synonymous QPCTL variant has also been identified as a top rare gene variant associated with opioid dependence (25). These studies provide evidence that genetic elements of QPCTL may impact other diseased states such as obesity or opioid dependence.

## QPCT/L in neuro-degenerative disorders

### Alzheimer’s disease

**Figure 1:**
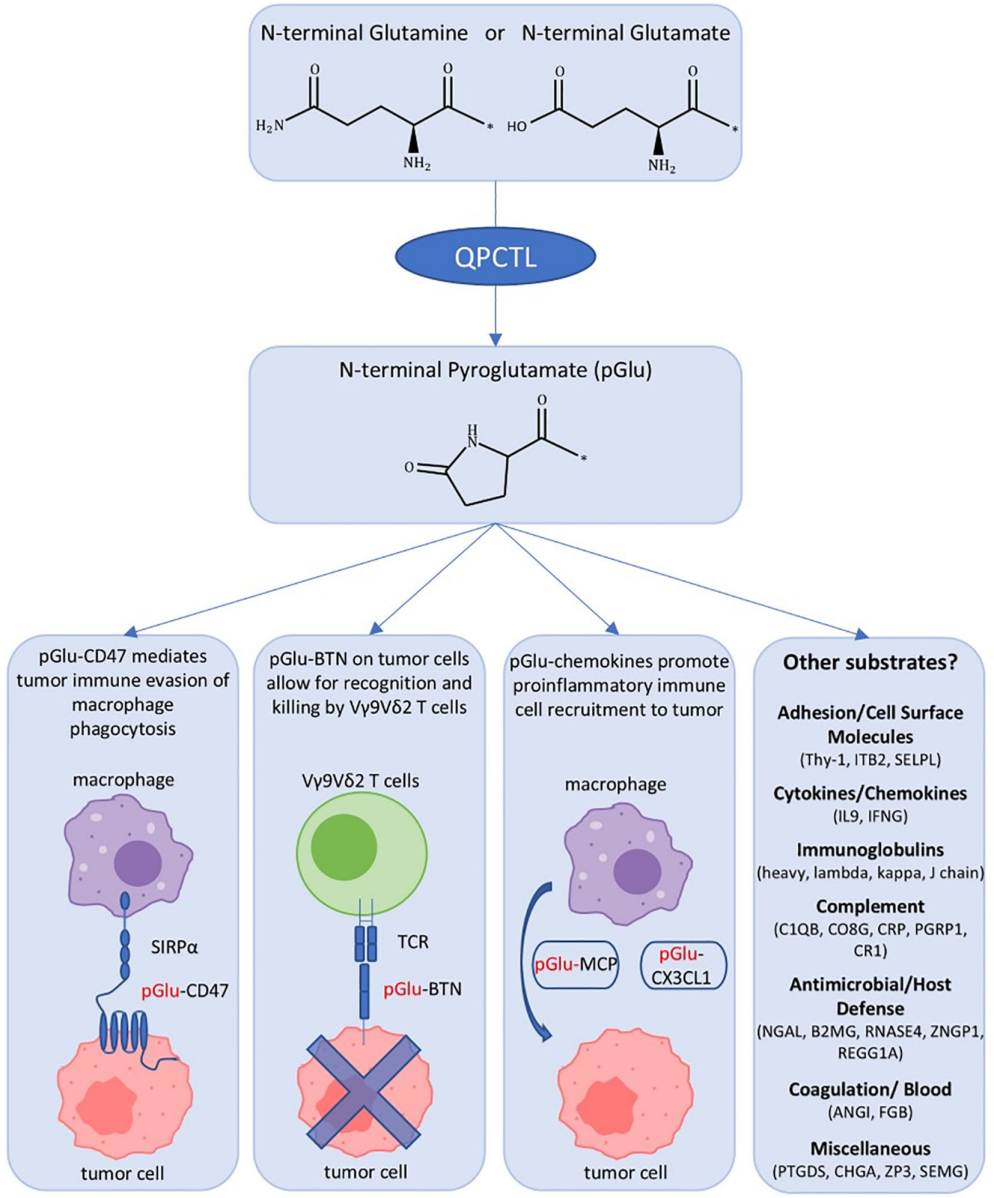
Overview of QPCTL impact on tumor cells and future directions in identifying further substrates. QPCTL catalyzes the formation of pyroglutamate (pGlu) on N-terminal glutamine or glutamate residues, which can have impacts on tumor immune evasion or recognition via various substrates. Immune cell-related proteins identified as predicted substrates for gluataminyl cyclases are listed. Abbreviations: pGlu, pyroglutamate; SIRPα, signal regulatory protein α; BTNs, butyrophilins; TCR, T cell receptor; MCP, monocyte chemoattractant protein; CX3CL1, fractalkine/chemokine ligand 1; other substrate definitions, see **Table 1**.

Alzheimer’s Disease (AD) is characterized by the formation of amyloid plaques due to the accumulation of amyloid-β (Aβ) peptides and neurofibrillary tangles. These aggregations caused by hyperphosphorylated Tau proteins in the brain result in neuronal damage and death (26). In AD, QPCT generates pyroglutamylated Aβ peptides which are highly prone to aggregation and the formation of pathogenic plaques (27, 28). Moreover, QPCTL was shown to modify the chemokine CCL2 (MCP-1). In turn, the secretion of pGlu-CCL2 by astrocytes fosters inflammatory processes by increasing the recruitment of monocytes and other immune cells to inflamed sites to the brain (29) (**Figure 1**, **Table 1**). As described later in this review, pGlu-CCL2 is also critically involved in shaping tumor microenvironments.

**Table 1:** Validated and predicted substrates for glutaminyl cyclases.

Functional group	Protein	Full protein name	Function (based on www.uniprot.org or published data)	Human	Mouse
Adhesion and Cell Surface Molecules	**CD47**	**Leukocyte surface antigen CD47**	Immune evasion by tumor cells mediated through the pGluCD47/SIRPα axis	Yes	Yes
	**BTN**	**Butyrophilins**	Immunoglobulin superfamily proteins with immunomodulatory functions, like phosphoantigen presentation to γδ T cells. Promotes killing of tumor cells	Yes	Yes (for some BTNs)
	THY1	Thy-1 membrane glycoprotein	May play a role in cell-cell or cell-ligand interactions during synaptogenesis and other events in the brain	Yes	Yes
	ITB2	Integrin beta-2	Involved in leukocyte adhesion and transmigration of leukocytes including T-cells and neutrophils	Yes	Yes
	SELPL	P-selectin glycoprotein ligand 1	Mediates rapid rolling of leukocytes over vascular surfaces during the initial steps in inflammation	Yes	Yes
	CD3ϵ	T cell surface glycoprotein CD3 epsilon chain	Part of the TCR-CD3 complex present on T lymphocyte cell surface	Yes	Yes
Cytokines/Chemokines	**CCL2**	**C-C motif chemokine 2**	Exhibits a chemotactic activity for monocytes and basophils but not neutrophils or eosinophils	Yes	Yes
	**CCL7**	**C-C motif chemokine 7**	Chemotactic factor that attracts monocytes and eosinophils, but not neutrophils	Yes	Yes
	**CX3CL1**	**Fractalkine**	Regulates leukocyte adhesion and migration processes at the endothelium	Yes	Yes
	**CCL8**	**C-C motif chemokine 8**	Chemokine attractant for monocytes, lymphocytes, basophils, and eosinophils	Yes	No
	**CCL13**	**C-C motif chemokine 13**	Chemotactic factor that attracts monocytes, lymphocytes, basophils and eosinophils, but not neutrophils	Yes	No
	IL9	Interleukin-9	Multifunctional cytokine secreted mainly by T-helper 2 lymphocytes and also mast cells or NKT cells that plays important roles in the immune response against parasites	Yes	Yes
	IFNG	Interferon gamma	Type II interferon produced by immune cells such as T-cells and NK cells that plays crucial roles in antimicrobial, antiviral, and antitumor responses by activating effector immune cells and enhancing antigen presentation	Yes	No
Hormones	**GnRH**	**gonadotropin-releasing hormone**	Produced in the hypothalamus, regulates the reproductive system through stimulation of the pituitary gland to release luteinizing hormone (LH) and follicle-stimulating hormone (FSH)	Yes	Yes
	**TRH**	**thyrotropin-releasing hormone**	Produced by the hypothalamus, stimulates the pituitary gland to produce thyroid-stimulating hormone (TSH)	Yes	Yes
Immunoglobulins	Ig Chains variable	Immunoglobulin chains variable (heavy, lambda, kappa)	Immunoglobulins are present as membrane-bound receptors (B cell receptors on the surface of B-lymphocytes) or as secreted glycoproteins (antibodies) produced by plasmablasts and plasma cells	Yes	Yes
	IGJ	Immunoglobulin J-chain	Serves to link monomer units of either IgM or IgA	Yes	No
Complement/Acute Phase	C1QB	Complement C1q subcomponent subunit B	Core component of the complement C1 complex, a multiprotein complex that initiates the classical pathway of the complement system	Yes	Yes
	CO8G	Complement component C8 gamma chain	Compenent of the membrane attack complex activated by the complement cascade, which leads to cell lysis	Yes	Yes
	CRP	C-reactive protein	Promotes agglutination, bacterial capsular swelling, phagocytosis and complement fixation through calcium-dependent binding to phosphorylcholine	Yes	No
	PGRP1	Peptidoglycan recognition protein 1	Innate immunity protein that plays several important functions in antimicrobial and antitumor defense systems	Yes	No
	CR1	Complement receptor type 1	Membrane immune adherence receptor that plays a critical role in the capture and clearance of complement-opsonized pathogens by erythrocytes and monocytes/macrophages	Yes	No
Antimicrobial/Host Defense	NGAL	Neutrophil gelatinase-associated lipocalin	Iron-trafficking protein involved in multiple processes such as apoptosis, innate immunity and renal development	Yes	Yes
	B2MG	Beta-2-microglobulin	Component of the class I major histocompatibility complex (MHC). Involved in the presentation of peptide antigens to the immune system	Yes	No
	RNASE4	Ribonuclease 4	Has antimicrobial activity against uropathogenic E.coli	Yes	Yes
	ZNGP1	Zinc-alpha-2-glycoprotein	Stimulates lipid degradation in adipocytes and causes extensive fat losses associated with some advanced cancers	Yes	Yes
	REG1A	Lithostathine-1-alpha	Might act as an inhibitor of spontaneous calcium carbonate precipitation. May be associated with neuronal sprouting in brain, and with brain and pancreas regeneration	Yes	Yes
Neuronal regulators	**APP**	**Amyloid-precursor protein, cleavage product: Amyloid-β**	Transmembrane protein in the nervous system, promotes neuronal development, synaptic plasticity, cell adhesion	Yes	Yes
	**HTT**	**Huntingtin**	Involved in intracellular transport, gene transcription, and survival. Transport of materials along nerve cell axons, regulating gene expression in neurons	Yes	Not determined
	**SNCA**	**α-synuclein**	Found in neuronal synapses, regulates the release of neurotransmitters	Yes	Yes
	NTS	Neurotensin	May play an endocrine or paracrine role in the regulation of fat metabolism. It causes contraction of smooth muscle	Yes	No
Coagulation/Blood	ANGI	Angiogenin	Secreted ribonuclease that can either promote or restrict cell proliferation of target cells, depending on the context	Yes	Yes
	FGB	Fibrinogen beta chain	Cleaved by the protease thrombin to yield monomers which, together with fibrinogen alpha (FGA) and fibrinogen gamma (FGG), polymerize to form an insoluble fibrin matrix	Yes	No
Miscellaneous	PTGDS	Prostaglandin-H2 D-isomerase	Catalyzes the conversion of PGH2 to PGD2, a prostaglandin involved in smooth muscle contraction/relaxation and a potent inhibitor of platelet aggregation	No	Yes
	CHGA	Chromogranin-A	Regulates granule biogenesis in endocrine cells by up-regulating the transcription of protease nexin 1 (SERPINE2) via a cAMP-PKA-SP1 pathway	Yes	Yes
	ZP3	Zona pellucida sperm-binding protein 3	Component of the zona pellucida, an extracellular matrix surrounding oocytes which mediates sperm binding, induction of the acrosome reaction and prevents post-fertilization polyspermy	Yes	Yes
	SEMG	Semenogelin-1	Participates in the formation of a gel matrix entrapping the accessory gland secretions and ejaculated spermatozoa	Yes	Yes

Each substrate is categorized into functional groups and further explained on function and mouse vs human expression for a pGlu modification based on the Uniprot database description or based on published data (12). Validated substrates labeled are in bold text, while predicted substrates are labeled in fine text.

In both a transgenic Tg2576 AD mouse model and human patients, QPCTL and CCL2 are upregulated (30). This leads to increased levels of pGlu-Aβ and pGlu-CCL2 which further induce inflammation and immune cell recruitment (29). Several inhibitors have been developed to interfere with QPCT-mediated pGlu-Aβ and QPCTL-mediated pGlu-CCL2 formation in AD. PQ912 (Varoglutamstat) is a small molecule inhibitor that blocks both glutaminyl cyclases (31). Treatment of early-stage AD patients in a phase 2b clinical trial with PQ912 unfortunately failed to improve patient cognitive functions and was therefore terminated (32). Thus, the development of new and refined QPCT/L inhibitors is a current challenge in AD drug development.

### Huntington’s disease

Huntington’s disease (HD) is a neurodegenerative disorder caused by a CAG trinucleotide expansion in exon 1 of the gene encoding for the Huntingtin protein (HTT). The mutant HTT protein contributes to the disease through aggregation triggered by the presence of a long N-terminal polyglutamine stretch (33). Through a broad siRNA-based knockdown approach in mammalian cells, Jimenez-Sanchez and colleagues identified QPCT as one of the strongest novel suppressors of HTT toxicity caused by mutant HTT proteins. Knockdown of QPCT protected against HTT toxicity in cell lines expressing human HTT variants. These findings were confirmed in a HD Drosophila model in which RNAi-mediated knockdown of either QPCT or QPCTL rescued eye depigmentation and the loss of photoreceptors as signs of neurodegeneration. However, the observed effects on HTT toxicity might be indirect as inhibition of QPCT resulted in an increase of the chaperone alpha-B crystallin which results in fewer HTT aggregates (34). QPCT inhibition might trigger stress responses, therefore, QPCT and potentially also QPCTL might modulate protein aggregation through the regulation of chaperones.

### Parkinson’s disease/human synucleinopathies

Parkinson’s disease (PD) is the second most frequent neurodegenerative disorder concomitant with the degeneration of dopaminergic neurons of the substantia nigra pars compacta, which in turn leads to dopamine depletion of the striatum. Loss or reduction of dopaminergic activity results in clinical symptoms such as hypokinesis and tremors (35). PD is characterized by the formation of Lewy bodies and Lewy neurites consisting of aggregated α-synuclein aggregates (36). Proteolytic cleavage of α-synuclein results in biologically active fragments, such as Gln79-α-synuclein that harbors a N-terminal glutamine and therefore constitutes a substrate for QPCT. Colocalization of QPCT and a-synuclein was detected in the mouse brain and moreover, pGlu-α-synuclein could be detected in the substantia nigra and in Lewy bodies and Lewy neurites in PD patients (37).

In summary, glutaminyl cyclases play a crucial role in the pathogenesis of neuro-degenerative diseases by modulating toxic peptide and protein aggregations as well as by promoting inflammation through the modification of chemokines such as CCL2.

## Immune evasion of tumor cells via the pGlu-CD47-SIRPα axis from macrophages

QPCTL has received significant attention in cancer research due to its role in regulating tumor-related processes. Overexpression of QPCTL is observed in various cancers, including renal cell carcinoma, melanoma, glioma, and papillary thyroid carcinoma, where its high levels are typically associated with poorer survival outcomes (38–40).

QPCTL has a major role in tumor immune evasion via the CD47-SIRPα axis, with the pyroglutamylated form of CD47 having higher binding affinity with SIRPα on macrophages (41). The CD47-SIRPα axis is a mechanism of which cancer cells evade phagocytosis of macrophages (**Figure 1**, **Table 1**). Tumor cells often also overexpress CD47, which binds to SIRPα on myeloid cells and transmits a “don’t eat me” signal (40, 42). Inhibiting the CD47-SIRPα axis aims to restore macrophage’s ability to properly phagocytose tumor cells, however, direct inhibition of CD47 can lead to the lysis of red blood cells and anemia. Indirect inhibition of the CD47-SIRPα axis by targeting QPCTL can circumvent this issue, since red blood cells do not contain Golgi apparatus or express QPCTL at all (43).

Current therapeutics in development aim to competitively inhibit glutaminyl cyclases. Due to the highly conserved active site, most inhibitors have the potential to inhibit both enzymes. Usually, they are more effective in inhibiting QPCT due to its more closed active site (44). It is crucial to fully characterize the efficacy and unknown impacts of QPCTL inhibition. Logtenberg and colleagues showed that inhibition of QPCTL by SEN177 reduced binding of an anti-pGlu-CD47 antibody in melanoma, epidermoid carcinoma, and Burkitt’s leukemia cells *in vitro.* Furthermore, QPCTL inhibition in combination with therapeutic antibodies effectively increased phagocytosis of Burkitt’s lymphoma Raji cells by macrophages and neutrophil-mediated cytotoxicity of epidermoid carcinoma cells (41).

In B-cell acute lymphoblastic leukemia (B-ALL), a recent study by Kowol and colleagues confirmed the impact of pGlu on CD47 at the molecular level by substituting the pGlu residue with alanine. The mutated CD47 without pGlu had impaired functional activity with reduced binding to SIRPα and binding to macrophages. Additionally, QPCTL inhibition by SEN177 in combination with therapeutic antibody anti-CD38 IgA2 improved antibody dependent cellular phagocytosis and cytotoxicity of REH cells. This study provides evidence for QPCTL inhibition as a promising immunotherapy for the treatment of B-ALL (45).

## Immune recognition of tumor cells via their pGlu-Butyrophilins by Vγ9Vδ2 T cells

Although it is well documented that QPCTL in cancer cells can lead to tumor immune evasion by modifying the CD47-SIRPα axis, emerging evidence shows that this is not true for all immune cell subtypes. In particular, loss of QPCTL in melanoma and leukemia cell lines impairs recognition and killing of cancer cells by Vγ9Vδ2 T cells (46). Vγ9Vδ2 T cells are the largest subpopulation of γδ T cells that target cancer and infectious cells by recognizing altered intracellular phosphoantigen (pAg) concentrations (47).

Vγ9Vδ2 T cells detect intracellular pAg concentrations in target cells via butyrophilins (BTNs), primarily BTN2A1 and BTN3A1 (48). BTNs consist of an extracellular domain with IgV and IgC (49). The extracellular IgV domain of BTN2A1 can interact directly with the Vγ9 T cell receptor (TCR) of Vγ9Vδ2 T cells (50).

Wu et al. identified that QPCTL-mediated pGlu-BTNs on cancer cells allow for recognition by Vγ9Vδ2 T cells. Through mass spectrometry analysis, pGlu modifications were detected in BTN2A1, BTN2A2 and BTN3A3. Knockdown of QPCTL reduced Vγ9Vδ2 T cell-mediated killing of K562 cells and the interactions with Vγ9Vδ2 T cells, in particular the TCR tetramer binding to cancer cells (46). Since BTNs contain the glutamine residue in the IgV domain and BTN2A1 interacts with this domain directly with the Vγ9 domain of Vγ9Vδ2 T cells, the pGlu modification could be important for this interaction (50). In summary, QPCTL plays an important role in the immune modulation for recognition and killing of cancer cells by Vγ9Vδ2 T cells.

QPCTL-mediated pGlu formation on CD47 and BTNs in cancer cells displays the varied impact of QPCTL on the tumor microenvironment, with pGlu-CD47 having pro-tumor and pGlu-BTNs having anti-tumor effects (**Figure 1**, **Table 1**). This raises the question of how QPCTL inhibition would impact tumors that express both pGlu-CD47 and pGlu-BTNs. Would the benefit of promoting macrophage phagocytosis via QPCTL inhibition of the CD47-SIRPα axis outweigh the risk of reducing Vγ9Vδ2 T cell-mediated killing? The answer may very likely depend on the tumor type and would have to be determined in future studies. Otherwise, one potential avenue to circumvent the issue could be combination therapy of QPCTL inhibition with BTNA agonists that promote the activated conformational form of BTNA and induce Vγ9Vδ2 T cell-mediated killing (51).

## Immune modulation mediated by pyroglutamylated chemokines

### Monocyte chemoattractant proteins

The binding of chemokines to their associated receptor on immune cells allows for migration towards a higher concentration of chemokines at an inflammation site (52). Chemokines of the CC family monocyte chemoattractant proteins (MCPs), which include CCL2, CCL7, CCL8, and CCL13, have high sequence homology with a conserved glutamine-proline motif at the N-terminus (53). *In vitro* experiments show that the pyroglutamated form of the MCPs are resistant to cleavage by dipeptidylpeptidase 4 (DPP4) that targets the glutamine-proline motif. The resistance to DPP4 cleavage has functional implications, with the pGlu-MCPs being more efficient in inducing receptor internalization on human THP-1 monocytes than the DPP4 cleaved MCP form (54). Additionally, QPCTL was identified to catalyze the formation of pGlu on CCL2 and CCL7 to protect them from DPP4-mediated inactivation *in vivo* (55). Thus, QPCTL could be a target for CCL2- or CCL7-mediated diseases (**Figure 1**, **Table 1**). A similar mechanism is likely possible for CCL8 and CCL13, however, it has not been studied *in vivo* yet.

The recruitment of monocytes into tumors via chemokine gradients can lead to the generation of tumor-associated macrophages that promote tumor immune evasion and are associated with poor patient outcomes (56). *In vivo*, da Silva and colleagues demonstrated that QPCTL promotes the migration of monocytes into tumors via pyroglutamylated CCL2 and CCL7, and that ablation of QPCTL altered the myeloid cell infiltration. QPCTL knockout mice showed reduced tumor growth by reducing pGlu-CCR2 and CCR7, reshaping the tumor myeloid cell infiltrate, and promoting a pro-inflammatory macrophage profile that enhanced T-cell activation. Additionally, combined treatment with a QPCTL inhibitor and anti-PD-L1 reduced tumor growth compared to each individual treatment alone (55). This supports the use of QPCTL inhibitors as a method of tumor suppression by targeting multiple pathways, including the reduced tumor evasion via blocking the CD47-SIRPα axis and the reduced functional activity of chemokines CCL2 and CCL7.

The infiltration of monocytes in chronic kidney disease (CKD) or non-alcoholic fatty liver disease (NAFLD) through the CCL2 chemokine gradient can promote inflammation and disease progression. Both an NFLAD mouse model and antibody-induced glomerulonephritis rat model treated with a glutaminyl cyclase inhibitor had reduced CCL2 and monocyte infiltration (57). Additionally, Vivoryon Therapeutics reported that the QPCT/L inhibitor varoglutamstat improved kidney function in patients with diabetes, although it failed to meet endpoints for Alzheimer’s disease treatment in the Phase 2 VIVIAD and VIV-MIND clinical trials (58). This suggests that pGlu modficiation on MCPs may impact both immune infiltration in tumor and kidney diseases, and provide glutaminyl cyclases as a potential immunotherapy target.

### CX3CL1

CX3CL1 (C-X3-C Motif Chemokine Ligand 1, fractalkine) mediates both adhesion and cell migration in inflammatory processes via binding to its corresponding receptor CX3CR1 (59). Kehlen and colleagues determined that both QPCTL and QPCT can catalyze the formation of pGlu on CX3CL1 *in vitro* (**Figure 1**, **Table 1**). In primary endothelial cells stimulated with proinflammatory cytokines TNF-α/IL-1β (HUVECs) QPCT was co-upregulated with CCL2 and ICAM1. The pyroglutamylated form of CX3CL1 was more efficient in inducing this upregulation of these genes and downstream signaling, which suggests that the during inflammation QPCT allows for the formation of fully active molecules (60). Again, this demonstrates that pyroglutamylated chemokines have the ability to induce immune cell migration and adhesion during inflammatory processes. CX3CL1-expressing tumor cells have been shown to recruit immune cells like T cells, natural killer cells, or dendritic cells that have anti-tumor effects (59). However, it is still unknown how the pyroglutamylation of this chemokine can affect anti-tumor immunity.

## The potential substrate spectrum of glutaminyl cyclases – new implications in immune regulation

With glutaminyl cyclase inhibitors currently in development for therapeutic use, it is crucial to identify substrates *in vivo* to understand their impact on immune cells in healthy and disease states. Due to their high homology, mouse models are a good avenue for research studies, however, some predicted substrates are only expressed in humans or are difficult to identify in database searches. Therefore, we have compiled a table of known and predicted substrates for glutaminyl cyclases with a focus on immunological related proteins and specified its potential as a substrate in human and/or mouse. Substrates were identified by searching the UniProt database for “pyrrolidone carboxylic acid” and by filtering for immune system processes gene ontology or based on published data. However, it is notable to point out that published substrates such as BTNs and CX3CL1 are not included in the results of this search, indicating that not all known pyroglutamylated proteins are annotated within the UniProt database. The following table therefore a focused selection of reviewed human and mouse proteins with the pyroglutamate residue related to the immune system.

Functional grouping of the substrates includes adhesion and cell surface molecules, cytokines/chemokines, hormones, immunoglobulins, complement/acute phase proteins, antimicrobial/host defense, neuronal regulators, coagulation/blood, and other miscellaneous proteins (**Figure 1**, **Table 1**). The first grouping of cell surface proteins includes known substrates of QPCTL that impact tumor progression including CD47 and BTNs (41, 46). In both of these cases, the QPCTL-mediated pGlu residue alters the binding affinity of the cell surface molecules and their subsequent downstream effects. A similar process could be possible for the leukocyte adhesion molecules Thy-1, ITB2, and SELPL (**Table 1**).

The next group of substrates are categorized as cytokines and chemokines. The majority of the research focuses on the monocyte chemoattractant proteins CCL2 and CCL7, and to a lesser extent CCL8 and CCL13. The pyroglutamylated version of MCPs has been shown to have increased resistance to protein degradation by DDP4, which enables prolonged signaling for attracting monocytes to the inflammation site (54, 55). Additionally, pyroglutamylated CX3CL1 upregulated adhesion and migration molecules more effectively than the non-post-translationally modified version (60). Newly identified predicted cytokine substrates that could also have their function impacted by pyroglutamylation include IL-9 and interferon-γ (**Table 1**).

In both human and mouse, the immunoglobulin variable regions for the heavy, kappa, and lambda chains predominantly contain an N-terminal glutamine or glutamate, providing a potential target for glutaminyl cyclases (61, 62) (**Figure 1**, **Table 1**). Interestingly, the spontaneous pyroglutamylation of monoclonal antibodies has been shown to alter the charge heterogeneity. This modification introduces a basic charge variant, resulting in a higher isoelectric point compared to the uncyclized form of N-terminal glutamic acid. Such changes in charge can affect the structural and functional properties of antibodies, potentially leading to alterations in their binding affinity and pharmacokinetics (63). Along this line, deletion of the QPCTL gene induced by CRISPR/Cas9 in LPS-stimulated mouse B cells *in vitro* resulted a reduction of IgM in cell culture supernatants (64). Further studies are necessary to fully characterize this effect and determine if it also translates to humans.

The human J-chain, which links monomeric immunoglobulin chains into pentameric IgM or dimeric IgA, has an N-terminal glutamate that can cyclize into pGlu (65). However, the pGlu modification is not possible on murine J-chain due to an N-terminal glycine residue (12). The Immgen Gene Skyline database reveals that splenic plasma cells display high QPCTL mRNA abundances that are generally five to ten times higher compared to the other immune cells (66). As such, the immunoglobulin heavy, kappa, lambda, and joining chain are potential substrates that may explain the target of QPCTL in plasma cells. However, it remains to be determined whether glutaminyl cyclases are involved in J-chain-mediated assembly of IgA1, IgA2 or IgM molecules in humans. These findings suggest a functional role of QPCTL in Ig assembly, secretion, and/or stability and an opportunity to determine if glutaminyl cyclases can catalyze the formation of pGlu on immunoglobulin chains *in vivo*. In addition, the presence of N-terminal pyroglutamate was also observed in the T cell receptor signaling transducer unit CD3ϵ with possible implications in targeting of CD3ϵ and TCR signaling (67, 68).

The remaining categories identified in the pyroglutamate database search revealed novel potential substrates of QPCT/L such as surface receptors and adhesion molecules (THY1, ITB2/Integrin beta 2), components of the complement system (C1QB, CO8G, CRP, PGRP1, CR1), antimicrobial/host defense (NGAL, B2MG, RNASE4, ZNGP1, REGG1A), coagulation (ANGI, FGB), and other miscellaneous proteins (PTGDS, CHGA, ZP3, SEMG) that have not yet been studied in this context (summarized in **Table 1**) (12). However, given evidence from already identified substrates, the potential post-translation modification of pGlu may impact the proteins binding affinity, stability, and functionality. Functional studies using QPCT/L inhibition, CRISPR/Cas9-mediated deletion or research employing QPCT/QPCTL-deficient mice will be instrumental to determine whether these proteins are modified by either QPCT or QPCTL. The functional impact of the pGlu modification for each substrate has to be determined and is beyond the scope of our review.

However, with the current research on QPCTL primarily focused on the CD47-SIRPα axis, it is important to highlight that QPCT/L with their huge spectrum of substrates may also affect hormonal regulations, tumor microenvironments and innate as well as adaptive immune responses. These aspects are of importance in the light of future therapeutical strategies that involve glutaminyl-cyclase inhibitors.
